# Geographic Variations in the Quality of Oral Anticoagulation With Vitamin K Antagonists in the Era of New Anticoagulants

**DOI:** 10.1161/JAHA.113.000088

**Published:** 2013-02-22

**Authors:** Laurent Fauchier, Sophie Taillandier

**Affiliations:** 1 Pôle Cœur Thorax Vasculaire, Centre Hospitalier Universitaire Trousseau, Tours, 37044, France (L.F., S.T.); 2 Faculté de Médecine, Université François Rabelais, Tours, 37032, France (L.F.)

**Keywords:** Editorials, anticoagulant, atrial fibrillation, guideline adherence

## Introduction

A number of new drugs that do not exhibit the limitations of vitamin K antagonists (VKA) have now been evaluated for oral anticoagulation (OAC). These include direct thrombin inhibitors (dabigatran) and direct factor Xa inhibitors (eg, rivaroxaban, apixaban). Recent studies in patients with atrial fibrillation (AF) found higher efficacy and significantly lower incidences of intracranial bleeding for new agents compared with warfarin. New anticoagulants (NOACs) add to the therapeutic options and offer a number of advantages over warfarin, including significant convenience for both the clinician and patient compared to VKAs.^1^ Thus, where an OAC is recommended, one of the NOACs should be considered instead of adjusted‐dose VKA (international normalized ratio [INR] 2.0 to 3.0) for most patients with AF. However, NOACS should not be used in many AF patients, including those with rheumatic valvular disease (predominantly mitral stenosis) or prosthetic heart valves, and those with severe kidney disease.^2^ Patients reluctant to receive a new drug will still be treated with warfarin. Although newer anticoagulants have important advantages over warfarin, they are also expensive and VKA will continue to be widely used worldwide. Where a VKA is used, stroke prevention is effective where the individual mean time in therapeutic range (TTR) is good and efforts to improve quality of INR‐control are needed in order to achieve high TTRs.^3–6^

In the Rivaroxaban Once Daily Oral Direct Factor Xa Inhibition Compared with Vitamin K Antagonism for Prevention of Stroke and Embolism Trial in Atrial Fibrillation (ROCKET AF)trial, rivaroxaban was associated with a 12% reduction in the incidence of stroke and systemic embolism, compared with warfarin.^7^ However, patients remained in the therapeutic range for INR only 55% of the time, which is less than in Randomized Evaluation of Long‐Term Anticoagulant Therapy (RE‐LY) (64%) and in the Apixaban for Reduction In Stroke and Other ThromboemboLic Events in Atrial Fibrillation (ARISTOTLE) trial (66%).^8^ This shorter time spent in therapeutic range has been a concern for the ROCKET‐AF trial, but could actually reflect what happens in real life. TTR is certainly not a relevant marker of the superiority of any NOAC over VKAs. The benefit of NOAC compared to warfarin is often observed irrespective of the level of INR, but its magnitude may be affected by TTR, which is largely a marker of local standard of care.^8^ In this issue, Singer et al^9^ report their findings that clinical features of the patients in ROCKET‐AF, such as heart failure, were significant but modest determinants of individual TTR (iTTR). A notable finding is the striking influence of geographic region (with average iTTR ranging from 36% to 64%) and presumably reflecting different levels of aggressiveness in achieving the INR target, different support systems to manage warfarin, and different regional barriers to frequent INR testing and warfarin dose changes. While the understanding of the determinants of iTTR is still incomplete, the study suggests that providers of care, and the systems in which they work, have a deep effect on the quality of anticoagulation.

A concern is that the study included a particular set of physicians. It is not clear that the practitioners selected as investigators in a major clinical trial are accurately representative of all physicians in each region. This may rather reflect how clinical research organizations locally select contributing institutions in a randomized trial. The authors indicate that investigators were chosen on the basis of past performance in clinical trials and based on their access to large clinical practices that included patients with AF. This statement captures how investigators are generally selected for very large randomized trials. The fact that the pattern of regional variation in TTR was very similar to that observed in other studies supports the opinion that most of the randomized trials in the field are done with common investigators in each country, selected by the same clinical research organizations. In the 13 countries where both the RE‐LY and ROCKET trials enrolled patients, the rank order of mean iTTR values by country appears highly correlated.^8–9^ Without random sampling of physicians in each country, one cannot affirm that the results are truly representative of anticoagulation management in each country/region. It is very unlikely that such a study using regional random sampling across many countries will be performed. The data from Sweden deserve consideration. In 3 major trials, Sweden was the best performing country.^4,7–8^ A recent analysis from a comprehensive registry of Swedish patients on anticoagulants reports a remarkably high value for mean national iTTR (74%) which is practically the same as the mean iTTR observed for Swedish patients in ROCKET (75%).^9–10^ At least for this country, where one has nationally representative measures of iTTR, there is excellent agreement between trial results and countrywide clinical care. Anyway, whether the findings in ROCKET AF are truly ascribable to countries and regions or simply to sets of investigators in those areas does not alter the main conclusion of the authors, which is that practice setting is a dominant determinant of anticoagulation quality.

As a matter of fact, we do not know if patients in each region were included in primary, secondary, or tertiary care, in private or public institutions, cardiology or noncardiology departments, or mainly with inpatient or outpatient selection. Anyhow, the reader should not be led to believe that TTR is lower in India and Eastern Europe because physicians and/or patients act unreasonably and are less efficient or reliable. The hypothesis that the influence of geographic region reflects different levels of aggressiveness in achieving the INR point target of 2.5, different support systems to manage warfarin, and different barriers to frequent INR testing and warfarin dose adjustment is not demonstrated point by point with the current analysis. It is highly possible that patients had different TTR because they were seen and followed differently. Regional groupings were modified from those used in the primary trial report and some of them are surprising. Australia and New Zealand were associated with Western Europe and not with Canada/United States while the latter would be more appropriate from an anthropologic point of view. Similarly, it seems strange seeing Greece included in Eastern Europe while Israel is with Western Europe. The goal was rather to aggregate countries into the most coherent regional groups in terms of patient, cultural, and health system features. This apparently resulted in more homogeneity of mean iTTR values in a given region. The authors indicate that even within regions, there was considerable variability across countries, supporting their main hypothesis that differences mainly reside in the organization of care.

One may propose that race might act as a confounding factor in such an analysis. While genetic polymorphisms certainly affect response to warfarin and do track with race,^11^ there is little evidence that these polymorphisms affect INR‐control after initiation of anticoagulation. There were strong regional differences in mean iTTR between Western Europe and Eastern Europe despite the fact that patients in both areas are predominantly white. Similarly, there were marked differences in mean iTTR between China and Hong Kong, where nearly all patients in both regions are of Asian race. Estimates for black participants were apparently unreported since white and Asian participants accounted for 95% of the ROCKET AF population.

Some physicians may implicitly target the low end of the target range of INR 2.0 to 3.0 and this may be seen in all countries and all regions. This point may reflect the general behavior of physicians rather than their region of origin, but the systems within which the providers of care work is likely to have a significant effect on the quality of anticoagulation. In ROCKET AF all investigators were instructed to aim for INR 2.5, range 2.0 to 3.0. In many studies of iTTR, “too low” INR values are the main explanation for low iTTR values. Additionnaly, the high‐risk patients are more likely to be undertreated (with INR <2.0) than overtreated (with INR >3.0).^3^ Cognitive dysfunction is common in elderly patients with AF and is related to less effective anticoagulation and more vascular events.^12^ This concern of cause or consequence is often related to lower INR targets for older patients with AF. The Japan Circulation Society guidelines recommend INR 1.6 to 2.6 for AF patients aged 70 years and older.^13^ Although Japan was not included in ROCKET AF, there is evidence that East Asian physicians aim for modestly lower INR. These differences in concern for bleeding may also explain the lower mean iTTR in some regions, particularly in East Asia.

To what extent does less frequent monitoring account for lower iTTR? More frequent testing allows more rapid dose adjustment and this may explain why higher mean iTTR values correlate with more frequent testing.^14–15^ Such variations are commonly seen in a general population, but are less likely in a study like the ROCKET‐AF trial with close and regular management. The authors do not provide the information that the monitoring was significantly less frequent in some regions and/or the reason for different frequencies of INR testing, but one may assume that support mechanisms to respond to out‐of‐range values and patient barriers to visiting the medical centers once again play a role in addition to clinical features.

Exploring these different patterns of anticoagulation management would be a logical next step. Key points to improve the quality of anticoagulation include avoiding the loss of follow‐up and poor patient adherence to therapy, as well as the promotion of patient education, the use of standard target INR ranges, timely follow‐up after deranged INR values, the occasional use of anticoagulation clinic, and a wider adoption of dose support algorithms.^15^ The algorithm‐based systems facilitating warfarin dosing optimize anticoagulation quality and may improve clinical outcomes in AF. Such a specific treatment algorithm for anticoagulation management was not provided in the ROCKET‐AF and this may explain why TTR was lower than in other trials. In addition, a recent analysis of data from the RE‐LY trial found that adherence—‐intentional or not—to a simple warfarin dosing algorithm predicts improved TTR and accounts for considerable TTR variation between centers and countries.^16^ Consequently, changing the dose of warfarin for any (even slightly) out‐of range INR is probably the best option.^15^ Taken together, it appears that a precise, easy, and well‐followed scheme is a preeminent way to improve the quality of anticoagulation in AF patients. As in many aspects of medicine, this reinforces the conviction that there is no need for each physician to reinvent the wheel in each care system.
